# Crystal structure of La_24_Ru_11_

**DOI:** 10.1107/S2056989020008695

**Published:** 2020-07-03

**Authors:** P. Cattaneo

**Affiliations:** a Sezione di Chimica Inorganica e Metallurgia - Dipartimento di Chimica e Chimica Industriale, Università degli Studi di Genova, Genova, Italy; b Centrum för Analys och Syntes - Kemicentrum, Lunds Universitet, Lund, Sweden

**Keywords:** crystal structure, ruthenium, lanthanum, hexa­gonal, triad, non-centrosymmetric, inter­metallic

## Abstract

The compound La_24_Ru_11_ (tetra­cosa­lanthanum undeca­ruthenium) crystallizes in a Ce_24_Co_11_-type structure. The non-centrosymmetric crystal structure (space group *P*6_3_ *mc*) contains RuLa_6_ trigonal prisms, La_6_ octa­hedra and LaRu_4_ tetra­hedra and is closely related to that of Ce_23_Ni_7_Mg_4_. This communication highlights the crystal-chemical similarities and points out the differences between the two structures. All of the tested crystals were inversion twins.

## Abstract 2

The binary hexa­gonal inter­metallic, La_24_Ru_11_, was synthesized and the structure determined as part of a study of the rare-earth-rich part of the La–Ru system.

## Chemical context   

The La–Ru system has been extensively studied (Palenzona & Cirafici, 1989 ▸). The phase diagram contains five binary phases: the Laves phase, LaRu_2_ (Compton & Matthias, 1959 ▸), with the MgCu_2_-type structure; La_5_Ru_3_ (Palenzona & Canepa, 1990*a* ▸); La_7_Ru_3_ (Palenzona & Canepa, 1990*b* ▸), with the Sr_7_Pt_3_-type structure; La_5_Ru_2_ (Palenzona, 1979 ▸), with the Mn_5_C_2_-type structure and La_3_Ru (Palenzona, 1979 ▸), with the cementite-type structure. According to a recent study (Carlsson, 2015 ▸), the phase La_5_Ru_3_ is believed to be a part of an incommensurate-composite-structure family related to Y_44_Ru_25_.

During a systematic search for optimal crystal-growth conditions for La_5_Ru_3_, the new compound, La_24_Ru_11_, was obtained as a secondary product. It crystallizes with a hexa­gonal unit cell, space group *P*6_3_ *mc* (186), and with a Ce_24_Co_11_ structure type (Larson & Cromer, 1962 ▸).

According to the Pearson’s Crystal Data (Villars & Cenzual, 2019 ▸), the composition ratio of 24:11 is not common and only a few binary compounds having this composition have been reported (Singh & Raman, 1968 ▸; Raevskaya *et al.*, 1994 ▸). However, there are several ternary inter­metallics with a rare-earth content higher than 60 at.%, including Yb_9_CuMg_4_ (De Negri *et al.*, 2016 ▸), La_43_Ni_17_Mg_5_ (Solokha *et al.*, 2009*a* ▸) and Ce_23_Ni_7_Mg_4_ (Solokha *et al.*, 2009*b* ▸), which share some structural features with the title compound, La_24_Ru_11_, described below.

## Structural commentary   

The hexa­gonal primitive structure of La_24_Ru_11_, containing 70 atoms per cell, was solved with data acquired by a single-crystal X-ray diffraction measurement using a charge-flipping algorithm (Oszlányi & Süto, 2004 ▸, 2005 ▸) in the *SUPERFLIP* program (Palatinus & Chapuis, 2007 ▸) implemented in the *JANA2006* package (Petříček *et al.*, 2014 ▸).

**Figure 1:**
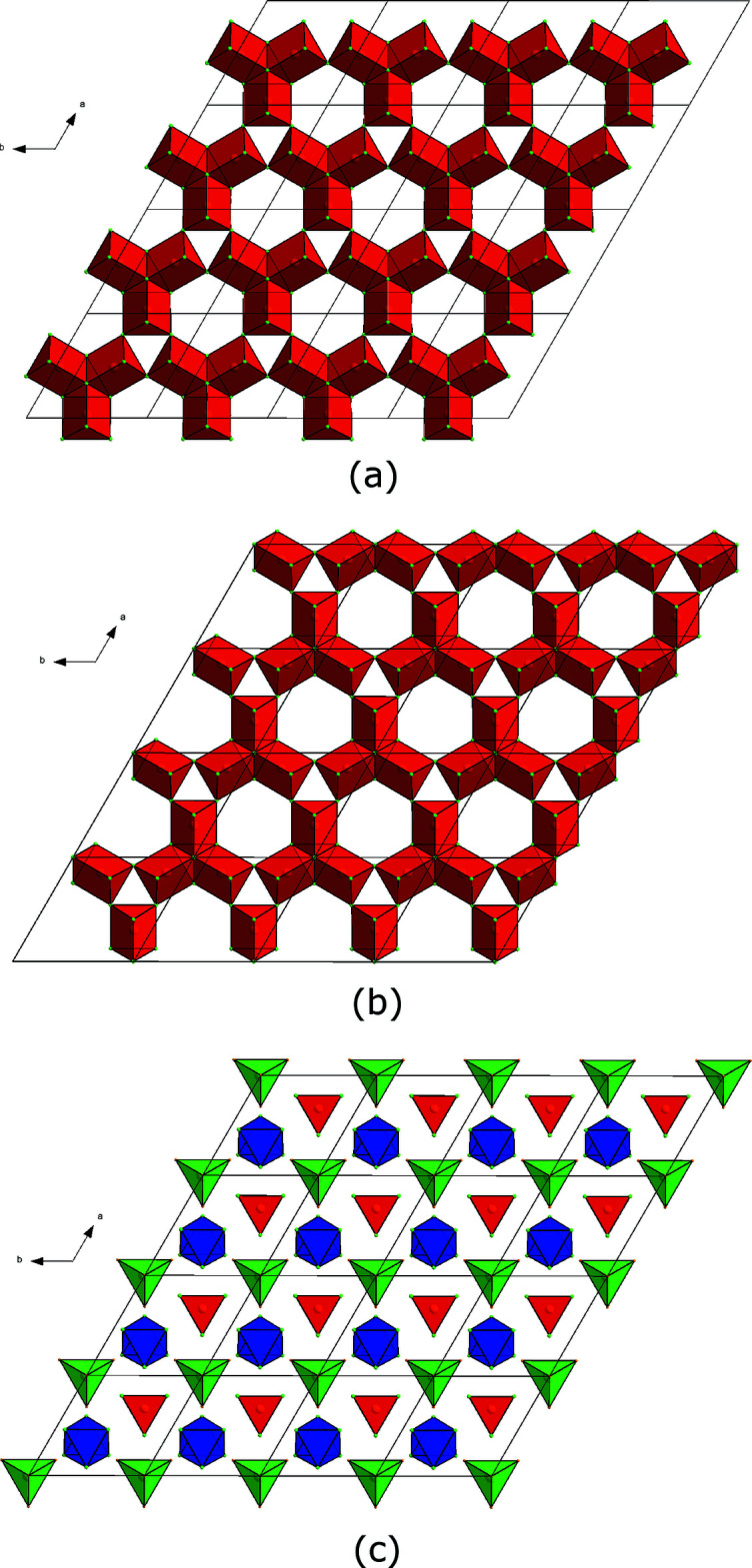
(*a*),(*b*) Distribution of triads of RuLa_6_ trigonal prisms (red) within slab *A* and slab B, respectively; (*c*) distribution of RuLa_6_ trigonal prisms (red), La_6_ octa­hedra (blue) and LaRu_4_ tetra­hedra (green) within slab *C*.

The structure is closely related to that of Ce_23_Ni_7_Mg_4_ (Solokha *et al.*, 2009*b* ▸) and can be described in terms of stacking along (00*z*) of the three different slabs *A*, *B* and *C* shown in Fig. 1 ▸(*a*), 1(*b*) and 1(*c*), respectively.

Slabs *A* and *B* are formed from trigonal prisms (consisting of six lanthanum atoms coordinated to a central ruthenium atom), three of which are joined together by sharing common edges and a vertex, to form triads. The two slabs are very similar to each other: slab *B* may be generated simply by rotating slab *A* by a 60° angle around the sixfold rotation axis of the lattice and translating it by the vector (2/3,2/3,0).

Structures containing only *A* and *B* slabs have previously been reported; for example, Ru_7_B_3_ (Hyde *et al.*, 1979 ▸) consists of an infinite packing of *ABAB* slabs in which the trigonal prisms are formed by ruthenium atoms coordinating to central boron atoms. About 50 isostructural binary compounds with general composition *R*_7_ *T*_3_, formed by a transition metal (*T*) with a lanthanide/actinide (*R*), have been discovered up to now and include Th_7_Fe_3_, Th_7_Co_3_ and Th_7_Ni_3_ (Palenzona & Cirafici, 1989 ▸), Nd_7_Pd_3_ (Moreau & Parthé, 1973 ▸) and Pr_7_Pd_3_ (Moreau & Parthé, 1973 ▸).

Slab *C* shown in Fig. 1 ▸(*c*) consists of three polyhedra: isolated Ru-centred trigonal prisms of lanthanum atoms (red), joining slabs *A* and *B* and oriented along the (00*z*) direction, empty La_6_ octa­hedra (blue) and La-centred ruthenium tetra­hedra (green). In both La_24_Ru_11_ and the related structure, Ce_23_Ni_7_Mg_4_ (Solokha *et al.*, 2009*b* ▸), the empty octa­hedra are formed by the rare-earth component. The compositional difference between these two structures arises from the the presence of an additional atom of La inside each ruthenium tetra­hedron in the title compound.

**Figure 2:**
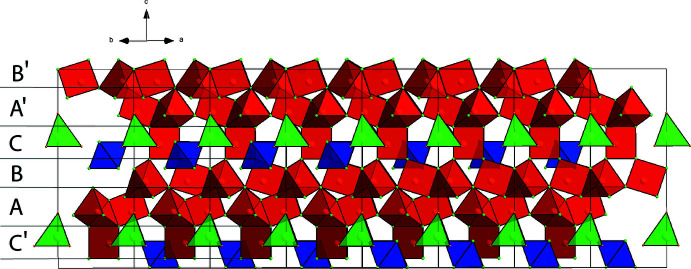
*ABCA*′*B*′*C*′ stacking of slabs formed by trigonal prisms (red), octa­hedra (blue) and tetra­hedra (green) in La_24_Ru_11_.

The final stacking sequence is *ABCA*′*B*′*C*′ (Fig. 2 ▸) where *A*′, *B*′ and *C*′ are the slabs *A*, *B* and *C*, respectively, rotated by a 60° angle around the sixfold rotation axis of the lattice.

## Synthesis and crystallization   

A sample weighing 0.5001 g and with nominal composition La_65_Ru_35_ was prepared from powdered metal constituents in stoichiometric amounts (*m*_La_ = 0.3593 g and *m*_Ru_ = 0.1408 g). The powders were weighed in a glovebox, mixed together and pressed into a pellet. The pellet was then arc-melted (necessary to obtain total melting, since the Ru–La system contains high-melting inter­metallics) in a low-pressure Ar chamber to prevent oxidation and then annealed for 10 days at 800°C. The alloy was crushed and a number of crystals were extracted and analysed. In addition to the title compound, La_24_Ru_11_, a small qu­antity of the phase LaRu_2_ (c*F*24-MgCu_2_) was also present.

## Refinement details   

**Figure 3:**
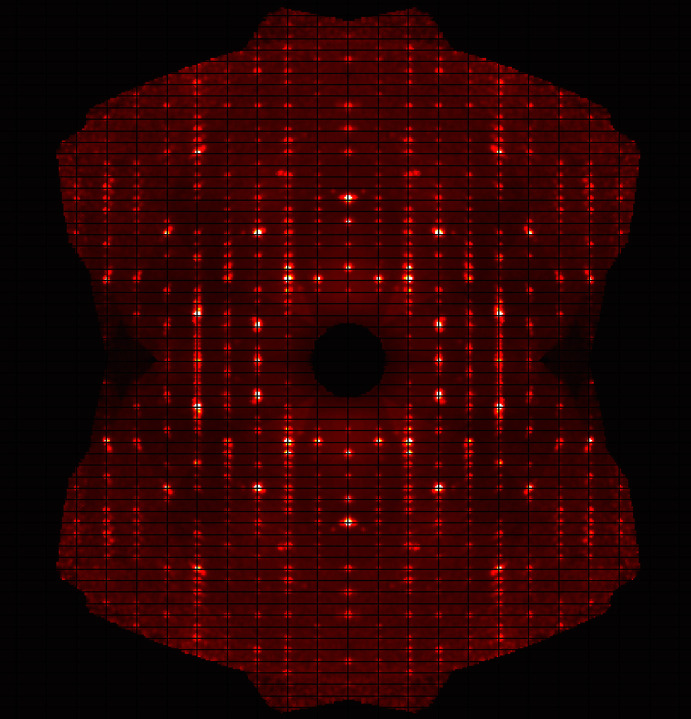
Diffraction pattern of the (0*kl*) layer showing twinned peaks and weak diffuse scattering, especially for the fifth-order reflections.

Crystal data, data collection and structure refinement details are summarized in Table 1 ▸. All of the tested crystals were twinnedby inversion, as confirmed by Flack-parameter refinement (Flack, 1983 ▸). In addition, a weak diffuse scattering in the diffraction pattern (probably due to stacking faults), is clearly visible in the (0*kl*) layer for fifth-order reflections (Fig. 3 ▸), which tend to overlap with their neighbours, forming streaks. This phenomenon is likely to be responsible for the slightly elevated values of residual electron density after the final refinement cycle. A B-C type 1 Gaussian isotropic extinction correction (Becker & Coppens, 1974*a* ▸,*b* ▸) was applied.

## Supplementary Material

Crystal structure: contains datablock(s) global, I. DOI: 10.1107/S2056989020008695/cq2037sup1.cif

Structure factors: contains datablock(s) global, I. DOI: 10.1107/S2056989020008695/cq2037Isup2.hkl

CCDC reference: 2012571

Additional supporting information:  crystallographic information; 3D view; checkCIF report

## Figures and Tables

**Table 1:** Experimental details

Crystal data	
Chemical formula	La_24_Ru_11_
*M*_r_	4445.9
Crystal system, space group	Hexagonal, *P*6_3_ *m* *c*
Temperature (K)	298
*a*, *c* (Å)	10.0627 (18), 22.801 (3)
*V* (Å^3^)	1999.5 (8)
*Z*	2
Radiation type	Mo *K*α
μ (mm^−1^)	29.00
Crystal size (mm)	0.23 × 0.2 × 0.2

Data collection	
Diffractometer	Rigaku Oxford Diffraction Xcalibur, Eos
Absorption correction	Analytical (*CrysAlis PRO*, Rigaku OD, 2019 ▸) [Analytical numeric absorption correction using a multifaceted crystal model based on Clark & Reid (1995 ▸)]
*T*_min_, *T*_max_	0.014, 0.059
No. of measured, independent and observed [*I* > 3σ(*I*)] reflections	6801, 1699, 1069
*R*_int_	0.094
(sin θ/λ)_max_ (Å^−1^)	0.646

Refinement	
*R*[*F* ^2^ > 2σ(*F* ^2^)], *wR*(*F*), *S*	0.057, 0.049, 1.24
No. of reflections	1069
No. of parameters	76
Δρ_max_, Δρ_min_ (e Å^−3^)	3.36, −3.12
Absolute structure	Flack (1983 ▸), 429 Friedel pairs used in the refinement
Absolute structure parameter	0.35 (9)

Computer programs: *CrysAlis PRO* (Rigaku OD, 2019 ▸), *JANA2006* (Petříček *et al.*, 2014 ▸) and *DIAMOND* (Brandenburg & Putz, 2019 ▸).

## supplementary crystallographic information

### Crystal data

**Table:**

La_24_Ru_11_	*D*_x_ = 7.385 Mg m^−^^3^
*M**_r_* = 4445.9	Mo *K*α radiation, λ = 0.71073 Å
Hexagonal, *P*6_3_*m**c*	Cell parameters from 1576 reflections
Hall symbol: P 6c -2c	θ = 4.8–27.3°
*a* = 10.0627 (18) Å	µ = 29.00 mm^−^^1^
*c* = 22.801 (3) Å	*T* = 298 K
*V* = 1999.5 (8) Å^3^	Hexagonal, grey
*Z* = 2	0.23 × 0.2 × 0.2 mm
*F*(000) = 3704	

### Data collection

**Table:**

Rigaku Oxford Diffraction Xcalibur, Eos diffractometer	1699 independent reflections
Radiation source: X-ray tube	1069 reflections with *I* > 3σ(*I*)
Graphite monochromator	*R*_int_ = 0.094
Detector resolution: 8.0683 pixels mm^-1^	θ_max_ = 27.3°, θ_min_ = 4.8°
ω scans	*h* = −6→11
Absorption correction: analytical (*CrysAlisPro*, Rigaku OD, 2019) [Analytical numeric absorption correction using a multifaceted crystal model based on Clark & Reid (1995)]	*k* = −13→10
*T*_min_ = 0.014, *T*_max_ = 0.059	*l* = −29→29
6801 measured reflections	

### Refinement

**Table:**

Refinement on *F*	Weighting scheme based on measured s.u.'s *w* = 1/(σ^2^(*F*) + 0.0001*F*^2^)
*R*[*F*^2^ > 2σ(*F*^2^)] = 0.057	(Δ/σ)_max_ = 0.0003
*wR*(*F*^2^) = 0.049	Δρ_max_ = 3.36 e Å^−^^3^
*S* = 1.24	Δρ_min_ = −3.12 e Å^−^^3^
1069 reflections	Extinction correction: B-C type 1 Gaussian isotropic (Becker & Coppens, 1974*a*,*b*)
76 parameters	Extinction coefficient: 100
0 restraints	Absolute structure: Flack (1983), 429 Friedel pairs used in the refinement
1 constraint	Absolute structure parameter: 0.35 (9)

### Special details

**Table:**

Refinement. Data collection and reduction were performed with CrysAlis PRO (Rigaku OD, 2019) software. Structure solution and refinement were performed with JANA2006 (Petříček *et al.*, 2014). During refinement, site occupation factors were checked, but no hints of disorder were found. In the final refinement cycles, all the atoms were refined with anisotropic thermal parameters. DIAMOND Version 4.6.3 (Brandenburg & Putz, 2019) was used for structure visualization and polyhedra construction.

### Fractional atomic coordinates and isotropic or equivalent isotropic displacement parameters (Å^2^)

**Table:**

	*x*	*y*	*z*	*U*_iso_*/*U*_eq_	
La1	1	1	0.6280 (3)	0.0285 (15)	
La2	0.45874 (16)	0.9175 (3)	0.6059 (3)	0.0274 (12)	
La3	0.5908 (4)	0.79539 (19)	0.4730 (3)	0.0270 (13)	
La4	0.87616 (17)	0.7523 (3)	0.8272 (3)	0.0274 (11)	
La5	0.4014 (3)	0.20071 (17)	0.2379 (3)	0.0300 (12)	
La6	0.79826 (19)	0.5965 (4)	0.5186 (3)	0.0262 (13)	
La7	0.20264 (16)	0.4053 (3)	0.1801 (3)	0.0298 (12)	
La8	0.333333	0.666667	0.3124 (3)	0.0260 (15)	
La9	0.9168 (3)	0.45838 (17)	0.3769 (3)	0.0274 (12)	
La10	1	1	0.4706 (3)	0.0299 (17)	
Ru1	0.8492 (3)	0.6983 (5)	0.4062 (3)	0.0301 (19)	
Ru2	0.666667	0.333333	0.5929 (3)	0.030 (2)	
Ru3	0.4838 (2)	0.5162 (2)	0.2522 (3)	0.0304 (17)	
Ru4	0.6909 (5)	0.8455 (3)	0.5825 (3)	0.0342 (19)	
Ru5	1	1	0.748776	0.039 (2)	

### Atomic displacement parameters (Å^2^)

**Table:**

	*U*^11^	*U*^22^	*U*^33^	*U*^12^	*U*^13^	*U*^23^
La1	0.031 (2)	0.031 (2)	0.0243 (17)	0.0153 (10)	0	0
La2	0.0274 (13)	0.0271 (19)	0.0276 (10)	0.0135 (9)	−0.0004 (6)	−0.0007 (12)
La3	0.029 (2)	0.0281 (15)	0.0241 (11)	0.0145 (10)	0.0004 (12)	0.0002 (6)
La4	0.0293 (13)	0.0287 (18)	0.0240 (9)	0.0144 (9)	0.0000 (6)	0.0000 (12)
La5	0.0307 (19)	0.0300 (13)	0.0296 (12)	0.0153 (9)	0.0067 (12)	0.0033 (6)
La6	0.0249 (15)	0.031 (2)	0.0245 (11)	0.0156 (11)	0.0007 (6)	0.0013 (11)
La7	0.0325 (15)	0.0271 (19)	0.0280 (11)	0.0136 (10)	0.0016 (6)	0.0032 (12)
La8	0.0189 (19)	0.0189 (19)	0.040 (2)	0.0095 (10)	0	0
La9	0.0267 (19)	0.0272 (14)	0.0281 (10)	0.0133 (10)	−0.0018 (12)	−0.0009 (6)
La10	0.031 (2)	0.031 (2)	0.0286 (19)	0.0153 (12)	0	0
Ru1	0.030 (2)	0.036 (3)	0.0264 (16)	0.0181 (15)	0.0007 (9)	0.0013 (17)
Ru2	0.030 (3)	0.030 (3)	0.028 (3)	0.0152 (15)	0	0
Ru3	0.034 (2)	0.034 (2)	0.0303 (16)	0.022 (2)	−0.0001 (8)	0.0001 (8)
Ru4	0.021 (3)	0.033 (3)	0.045 (2)	0.0104 (13)	−0.0056 (18)	−0.0028 (9)
Ru5	0.046 (3)	0.046 (3)	0.025 (3)	0.0228 (16)	0	0

### Geometric parameters (Å, º)

**Table:**

La1—La7^i^	3.727 (5)	La4—La10^xii^	3.918 (8)
La1—La7^ii^	3.727 (5)	La4—Ru1^viii^	3.023 (8)
La1—La7^iii^	3.727 (5)	La4—Ru1^xiii^	3.023 (7)
La1—La10	3.587 (11)	La4—Ru5	2.803 (5)
La1—Ru4	2.886 (6)	La5—La7	3.754 (5)
La1—Ru4^iv^	2.886 (6)	La5—La7^xiv^	3.754 (4)
La1—Ru4^v^	2.886 (6)	La5—La9^x^	3.885 (8)
La1—Ru5	2.755 (8)	La5—La9^xv^	3.885 (8)
La2—La2^vi^	3.786 (4)	La5—Ru3	2.871 (3)
La2—La2^vii^	3.786 (4)	La5—Ru3^x^	2.871 (5)
La2—La3	3.754 (8)	La6—La7^i^	3.683 (9)
La2—La3^vi^	3.754 (8)	La6—La9	3.932 (8)
La2—La5^i^	3.758 (8)	La6—La9^x^	3.932 (8)
La2—La5^viii^	3.758 (8)	La6—La10	3.682 (5)
La2—La6^vi^	3.748 (7)	La6—Ru1	2.713 (9)
La2—La6^v^	3.748 (6)	La6—Ru2	2.852 (7)
La2—La7^viii^	3.596 (7)	La7—La7^vi^	3.945 (4)
La2—La7^iii^	3.596 (5)	La7—La7^vii^	3.945 (4)
La2—Ru4	2.822 (6)	La7—La8	3.780 (8)
La2—Ru4^vi^	2.822 (4)	La7—Ru2^xvi^	3.024 (7)
La3—La3^vi^	3.886 (5)	La7—Ru3	2.965 (6)
La3—La3^vii^	3.886 (4)	La7—Ru3^vii^	2.965 (6)
La3—La4^ix^	3.610 (9)	La8—La9^xvii^	3.916 (5)
La3—La6	3.691 (6)	La8—La9^x^	3.916 (5)
La3—La6^v^	3.691 (4)	La8—La9^v^	3.916 (5)
La3—La9^x^	3.856 (6)	La8—Ru3	2.960 (5)
La3—La9^v^	3.856 (7)	La8—Ru3^vi^	2.960 (5)
La3—La10	3.567 (4)	La8—Ru3^vii^	2.960 (5)
La3—Ru4	2.644 (9)	La9—La9^x^	3.775 (5)
La4—La4^iv^	3.738 (4)	La9—La9^xv^	3.775 (4)
La4—La4^v^	3.738 (5)	La9—Ru1	2.895 (6)
La4—La5^i^	3.673 (6)	La9—Ru1^xv^	2.895 (4)
La4—La5^ii^	3.673 (6)	La9—Ru3^xv^	3.016 (9)
La4—La7^i^	3.623 (9)	La10—Ru1	3.012 (6)
La4—La8^i^	3.667 (4)	La10—Ru1^iv^	3.012 (6)
La4—La9^xi^	3.813 (5)	La10—Ru1^v^	3.012 (7)
La4—La9^viii^	3.813 (4)		
			
La7^i^—La1—La7^ii^	110.31 (14)	La2^xviii^—La7—La7^vi^	56.73 (9)
La7^i^—La1—La7^iii^	110.31 (14)	La2^xviii^—La7—La7^vii^	108.92 (11)
La7^i^—La1—La10	108.62 (15)	La2^xviii^—La7—La8	107.30 (10)
La7^i^—La1—Ru4	70.89 (10)	La2^xviii^—La7—Ru2^xvi^	65.98 (11)
La7^i^—La1—Ru4^iv^	70.89 (11)	La2^xviii^—La7—Ru3	63.25 (15)
La7^i^—La1—Ru4^v^	177.6 (3)	La2^xviii^—La7—Ru3^vii^	152.50 (10)
La7^i^—La1—Ru5	71.38 (15)	La4^xvi^—La7—La5	59.68 (13)
La7^ii^—La1—La7^iii^	110.31 (14)	La4^xvi^—La7—La5^xxi^	59.68 (12)
La7^ii^—La1—La10	108.62 (15)	La4^xvi^—La7—La6^xvi^	157.48 (12)
La7^ii^—La1—Ru4	177.6 (3)	La4^xvi^—La7—La7^vi^	109.16 (17)
La7^ii^—La1—Ru4^iv^	70.89 (11)	La4^xvi^—La7—La7^vii^	109.16 (18)
La7^ii^—La1—Ru4^v^	70.89 (14)	La4^xvi^—La7—La8	59.33 (13)
La7^ii^—La1—Ru5	71.38 (15)	La4^xvi^—La7—Ru2^xvi^	153.4 (2)
La7^iii^—La1—La10	108.62 (15)	La4^xvi^—La7—Ru3	67.10 (16)
La7^iii^—La1—Ru4	70.89 (12)	La4^xvi^—La7—Ru3^vii^	67.10 (17)
La7^iii^—La1—Ru4^iv^	177.6 (3)	La5—La7—La5^xxi^	107.61 (15)
La7^iii^—La1—Ru4^v^	70.89 (15)	La5—La7—La6^xvi^	110.42 (16)
La7^iii^—La1—Ru5	71.38 (15)	La5—La7—La7^vi^	90.45 (9)
La10—La1—Ru4	68.94 (19)	La5—La7—La7^vii^	144.57 (14)
La10—La1—Ru4^iv^	68.9 (2)	La5—La7—La8	90.35 (15)
La10—La1—Ru4^v^	68.94 (19)	La5—La7—Ru2^xvi^	126.05 (10)
La10—La1—Ru5	180	La5—La7—Ru3	48.87 (9)
Ru4—La1—Ru4^iv^	107.8 (2)	La5—La7—Ru3^vii^	125.3 (2)
Ru4—La1—Ru4^v^	107.8 (2)	La5^xxi^—La7—La6^xvi^	110.42 (14)
Ru4—La1—Ru5	111.06 (19)	La5^xxi^—La7—La7^vi^	144.57 (17)
Ru4^iv^—La1—Ru4^v^	107.8 (2)	La5^xxi^—La7—La7^vii^	90.45 (11)
Ru4^iv^—La1—Ru5	111.1 (2)	La5^xxi^—La7—La8	90.35 (15)
Ru4^v^—La1—Ru5	111.06 (19)	La5^xxi^—La7—Ru2^xvi^	126.05 (13)
La2^vi^—La2—La2^vii^	60.00 (9)	La5^xxi^—La7—Ru3	125.3 (2)
La2^vi^—La2—La3	90.76 (14)	La5^xxi^—La7—Ru3^vii^	48.87 (9)
La2^vi^—La2—La3^vi^	59.72 (13)	La6^xvi^—La7—La7^vi^	90.21 (14)
La2^vi^—La2—La5^i^	91.66 (14)	La6^xvi^—La7—La7^vii^	90.21 (16)
La2^vi^—La2—La5^viii^	59.76 (12)	La6^xvi^—La7—La8	143.19 (13)
La2^vi^—La2—La6^vi^	107.90 (12)	La6^xvi^—La7—Ru2^xvi^	49.12 (15)
La2^vi^—La2—La6^v^	146.9 (2)	La6^xvi^—La7—Ru3	123.75 (16)
La2^vi^—La2—La7^viii^	108.92 (12)	La6^xvi^—La7—Ru3^vii^	123.75 (18)
La2^vi^—La2—La7^iii^	150.8 (2)	La7^vi^—La7—La7^vii^	60.00 (9)
La2^vi^—La2—Ru4	106.59 (12)	La7^vi^—La7—La8	58.54 (12)
La2^vi^—La2—Ru4^vi^	47.87 (14)	La7^vi^—La7—Ru2^xvi^	49.28 (13)
La2^vii^—La2—La3	59.72 (13)	La7^vi^—La7—Ru3	48.29 (11)
La2^vii^—La2—La3^vi^	90.76 (15)	La7^vi^—La7—Ru3^vii^	95.77 (13)
La2^vii^—La2—La5^i^	59.76 (13)	La7^vii^—La7—La8	58.54 (12)
La2^vii^—La2—La5^viii^	91.66 (14)	La7^vii^—La7—Ru2^xvi^	49.28 (13)
La2^vii^—La2—La6^vi^	146.9 (2)	La7^vii^—La7—Ru3	95.77 (12)
La2^vii^—La2—La6^v^	107.90 (13)	La7^vii^—La7—Ru3^vii^	48.29 (12)
La2^vii^—La2—La7^viii^	150.8 (2)	La8—La7—Ru2^xvi^	94.06 (15)
La2^vii^—La2—La7^iii^	108.92 (12)	La8—La7—Ru3	50.31 (12)
La2^vii^—La2—Ru4	47.87 (9)	La8—La7—Ru3^vii^	50.31 (12)
La2^vii^—La2—Ru4^vi^	106.59 (14)	Ru2^xvi^—La7—Ru3	96.82 (12)
La3—La2—La3^vi^	62.33 (14)	Ru2^xvi^—La7—Ru3^vii^	96.82 (15)
La3—La2—La5^i^	107.05 (13)	Ru3—La7—Ru3^vii^	100.0 (2)
La3—La2—La5^viii^	147.62 (11)	La4^xvi^—La8—La4^ix^	119.17 (9)
La3—La2—La6^vi^	91.87 (18)	La4^xvi^—La8—La4^xxii^	119.17 (7)
La3—La2—La6^v^	58.94 (11)	La4^xvi^—La8—La7	58.21 (13)
La3—La2—La7^viii^	149.28 (18)	La4^xvi^—La8—La7^vi^	111.92 (15)
La3—La2—La7^iii^	106.97 (12)	La4^xvi^—La8—La7^vii^	111.92 (14)
La3—La2—Ru4	44.68 (17)	La4^xvi^—La8—La9^xvii^	60.27 (7)
La3—La2—Ru4^vi^	106.2 (2)	La4^xvi^—La8—La9^x^	60.27 (7)
La3^vi^—La2—La5^i^	147.62 (11)	La4^xvi^—La8—La9^v^	152.7 (2)
La3^vi^—La2—La5^viii^	107.05 (10)	La4^xvi^—La8—Ru3	66.52 (8)
La3^vi^—La2—La6^vi^	58.94 (13)	La4^xvi^—La8—Ru3^vi^	157.6 (3)
La3^vi^—La2—La6^v^	91.87 (18)	La4^xvi^—La8—Ru3^vii^	66.52 (9)
La3^vi^—La2—La7^viii^	106.97 (14)	La4^ix^—La8—La4^xxii^	119.17 (9)
La3^vi^—La2—La7^iii^	149.28 (18)	La4^ix^—La8—La7	111.92 (15)
La3^vi^—La2—Ru4	106.2 (2)	La4^ix^—La8—La7^vi^	58.21 (13)
La3^vi^—La2—Ru4^vi^	44.68 (17)	La4^ix^—La8—La7^vii^	111.92 (15)
La5^i^—La2—La5^viii^	64.37 (14)	La4^ix^—La8—La9^xvii^	152.7 (2)
La5^i^—La2—La6^vi^	152.73 (16)	La4^ix^—La8—La9^x^	60.27 (8)
La5^i^—La2—La6^v^	108.92 (11)	La4^ix^—La8—La9^v^	60.27 (10)
La5^i^—La2—La7^viii^	96.02 (19)	La4^ix^—La8—Ru3	66.52 (10)
La5^i^—La2—La7^iii^	61.34 (11)	La4^ix^—La8—Ru3^vi^	66.52 (10)
La5^i^—La2—Ru4	65.26 (18)	La4^ix^—La8—Ru3^vii^	157.6 (3)
La5^i^—La2—Ru4^vi^	126.9 (2)	La4^xxii^—La8—La7	111.92 (15)
La5^viii^—La2—La6^vi^	108.92 (13)	La4^xxii^—La8—La7^vi^	111.92 (15)
La5^viii^—La2—La6^v^	152.73 (16)	La4^xxii^—La8—La7^vii^	58.21 (13)
La5^viii^—La2—La7^viii^	61.34 (13)	La4^xxii^—La8—La9^xvii^	60.27 (7)
La5^viii^—La2—La7^iii^	96.02 (19)	La4^xxii^—La8—La9^x^	152.7 (2)
La5^viii^—La2—Ru4	126.9 (2)	La4^xxii^—La8—La9^v^	60.27 (9)
La5^viii^—La2—Ru4^vi^	65.26 (17)	La4^xxii^—La8—Ru3	157.6 (3)
La6^vi^—La2—La6^v^	64.01 (12)	La4^xxii^—La8—Ru3^vi^	66.52 (9)
La6^vi^—La2—La7^viii^	60.16 (15)	La4^xxii^—La8—Ru3^vii^	66.52 (10)
La6^vi^—La2—La7^iii^	94.82 (11)	La7—La8—La7^vi^	62.92 (13)
La6^vi^—La2—Ru4	123.6 (2)	La7—La8—La7^vii^	62.92 (13)
La6^vi^—La2—Ru4^vi^	62.57 (15)	La7—La8—La9^xvii^	91.15 (10)
La6^v^—La2—La7^viii^	94.82 (11)	La7—La8—La9^x^	91.15 (11)
La6^v^—La2—La7^iii^	60.16 (13)	La7—La8—La9^v^	149.09 (19)
La6^v^—La2—Ru4	62.57 (12)	La7—La8—Ru3	50.42 (12)
La6^v^—La2—Ru4^vi^	123.6 (2)	La7—La8—Ru3^vi^	99.4 (2)
La7^viii^—La2—La7^iii^	66.54 (11)	La7—La8—Ru3^vii^	50.42 (13)
La7^viii^—La2—Ru4	140.14 (15)	La7^vi^—La8—La7^vii^	62.92 (14)
La7^viii^—La2—Ru4^vi^	73.63 (16)	La7^vi^—La8—La9^xvii^	149.09 (19)
La7^iii^—La2—Ru4	73.63 (12)	La7^vi^—La8—La9^x^	91.15 (11)
La7^iii^—La2—Ru4^vi^	140.1 (2)	La7^vi^—La8—La9^v^	91.15 (11)
Ru4—La2—Ru4^vi^	146.0 (2)	La7^vi^—La8—Ru3	50.42 (13)
La2—La3—La2^vii^	60.56 (13)	La7^vi^—La8—Ru3^vi^	50.42 (14)
La2—La3—La3^vi^	58.83 (13)	La7^vi^—La8—Ru3^vii^	99.4 (2)
La2—La3—La3^vii^	89.24 (13)	La7^vii^—La8—La9^xvii^	91.15 (11)
La2—La3—La4^ix^	149.65 (12)	La7^vii^—La8—La9^x^	149.09 (19)
La2—La3—La6	109.8 (2)	La7^vii^—La8—La9^v^	91.15 (10)
La2—La3—La6^v^	60.44 (12)	La7^vii^—La8—Ru3	99.4 (2)
La2—La3—La9^x^	146.71 (12)	La7^vii^—La8—Ru3^vi^	50.42 (12)
La2—La3—La9^v^	90.79 (13)	La7^vii^—La8—Ru3^vii^	50.42 (12)
La2—La3—La10	108.59 (16)	La9^xvii^—La8—La9^x^	106.79 (13)
La2—La3—Ru4	48.63 (17)	La9^xvii^—La8—La9^v^	106.79 (13)
La2^vii^—La3—La3^vi^	89.24 (14)	La9^xvii^—La8—Ru3	125.78 (6)
La2^vii^—La3—La3^vii^	58.83 (12)	La9^xvii^—La8—Ru3^vi^	125.78 (8)
La2^vii^—La3—La4^ix^	149.65 (13)	La9^xvii^—La8—Ru3^vii^	49.67 (15)
La2^vii^—La3—La6	60.44 (12)	La9^x^—La8—La9^v^	106.79 (14)
La2^vii^—La3—La6^v^	109.79 (19)	La9^x^—La8—Ru3	49.67 (15)
La2^vii^—La3—La9^x^	90.79 (12)	La9^x^—La8—Ru3^vi^	125.78 (7)
La2^vii^—La3—La9^v^	146.71 (16)	La9^x^—La8—Ru3^vii^	125.78 (7)
La2^vii^—La3—La10	108.59 (18)	La9^v^—La8—Ru3	125.78 (7)
La2^vii^—La3—Ru4	48.63 (12)	La9^v^—La8—Ru3^vi^	49.67 (15)
La3^vi^—La3—La3^vii^	60.00 (10)	La9^v^—La8—Ru3^vii^	125.78 (9)
La3^vi^—La3—La4^ix^	109.74 (18)	Ru3—La8—Ru3^vi^	100.20 (19)
La3^vi^—La3—La6	146.80 (13)	Ru3—La8—Ru3^vii^	100.20 (19)
La3^vi^—La3—La6^v^	90.67 (12)	Ru3^vi^—La8—Ru3^vii^	100.20 (18)
La3^vi^—La3—La9^x^	108.14 (12)	La3^iv^—La9—La3^xv^	60.51 (11)
La3^vi^—La3—La9^v^	59.75 (11)	La3^iv^—La9—La4^xxiii^	106.19 (11)
La3^vi^—La3—La10	149.99 (8)	La3^iv^—La9—La4^xviii^	56.16 (12)
La3^vi^—La3—Ru4	106.6 (2)	La3^iv^—La9—La5^x^	153.45 (15)
La3^vii^—La3—La4^ix^	109.74 (17)	La3^iv^—La9—La5^xv^	112.04 (11)
La3^vii^—La3—La6	90.67 (10)	La3^iv^—La9—La6	56.57 (10)
La3^vii^—La3—La6^v^	146.80 (17)	La3^iv^—La9—La6^xv^	87.60 (17)
La3^vii^—La3—La9^x^	59.75 (10)	La3^iv^—La9—La8^xxiv^	67.06 (11)
La3^vii^—La3—La9^v^	108.14 (14)	La3^iv^—La9—La9^x^	108.14 (13)
La3^vii^—La3—La10	149.99 (12)	La3^iv^—La9—La9^xv^	144.6 (2)
La3^vii^—La3—Ru4	106.61 (18)	La3^iv^—La9—Ru1	61.43 (12)
La4^ix^—La3—La6	93.92 (16)	La3^iv^—La9—Ru1^xv^	118.7 (2)
La4^ix^—La3—La6^v^	93.92 (13)	La3^iv^—La9—Ru3^xv^	108.56 (12)
La4^ix^—La3—La9^x^	61.31 (13)	La3^xv^—La9—La4^xxiii^	56.16 (13)
La4^ix^—La3—La9^v^	61.31 (14)	La3^xv^—La9—La4^xviii^	106.19 (11)
La4^ix^—La3—La10	66.18 (15)	La3^xv^—La9—La5^x^	112.04 (13)
La4^ix^—La3—Ru4	137.77 (17)	La3^xv^—La9—La5^xv^	153.45 (16)
La6—La3—La6^v^	111.18 (14)	La3^xv^—La9—La6	87.60 (17)
La6—La3—La9^x^	62.74 (12)	La3^xv^—La9—La6^xv^	56.57 (12)
La6—La3—La9^v^	152.51 (17)	La3^xv^—La9—La8^xxiv^	67.06 (12)
La6—La3—La10	60.95 (8)	La3^xv^—La9—La9^x^	144.6 (2)
La6—La3—Ru4	64.68 (17)	La3^xv^—La9—La9^xv^	108.14 (13)
La6^v^—La3—La9^x^	152.51 (17)	La3^xv^—La9—Ru1	118.7 (2)
La6^v^—La3—La9^v^	62.74 (12)	La3^xv^—La9—Ru1^xv^	61.43 (15)
La6^v^—La3—La10	60.95 (9)	La3^xv^—La9—Ru3^xv^	108.56 (15)
La6^v^—La3—Ru4	64.68 (14)	La4^xxiii^—La9—La4^xviii^	112.06 (14)
La9^x^—La3—La9^v^	109.2 (2)	La4^xxiii^—La9—La5^x^	56.99 (11)
La9^x^—La3—La10	95.88 (13)	La4^xxiii^—La9—La5^xv^	108.06 (19)
La9^x^—La3—Ru4	124.98 (17)	La4^xxiii^—La9—La6	141.9 (2)
La9^v^—La3—La10	95.88 (11)	La4^xxiii^—La9—La6^xv^	87.10 (13)
La9^v^—La3—Ru4	124.98 (14)	La4^xxiii^—La9—La8^xxiv^	56.63 (7)
La10—La3—Ru4	71.60 (18)	La4^xxiii^—La9—La9^x^	145.54 (13)
La3^xiii^—La4—La4^iv^	109.74 (17)	La4^xxiii^—La9—La9^xv^	89.72 (9)
La3^xiii^—La4—La4^v^	109.74 (19)	La4^xxiii^—La9—Ru1	162.55 (11)
La3^xiii^—La4—La5^i^	123.63 (14)	La4^xxiii^—La9—Ru1^xv^	51.37 (15)
La3^xiii^—La4—La5^ii^	123.63 (15)	La4^xxiii^—La9—Ru3^xv^	64.02 (14)
La3^xiii^—La4—La7^i^	134.77 (12)	La4^xviii^—La9—La5^x^	108.06 (19)
La3^xiii^—La4—La8^i^	72.32 (14)	La4^xviii^—La9—La5^xv^	56.99 (11)
La3^xiii^—La4—La9^xi^	62.53 (15)	La4^xviii^—La9—La6	87.10 (12)
La3^xiii^—La4—La9^viii^	62.53 (13)	La4^xviii^—La9—La6^xv^	141.95 (19)
La3^xiii^—La4—La10^xii^	56.38 (13)	La4^xviii^—La9—La8^xxiv^	56.63 (8)
La3^xiii^—La4—Ru1^viii^	63.92 (19)	La4^xviii^—La9—La9^x^	89.72 (11)
La3^xiii^—La4—Ru1^xiii^	63.92 (16)	La4^xviii^—La9—La9^xv^	145.54 (16)
La3^xiii^—La4—Ru5	152.59 (18)	La4^xviii^—La9—Ru1	51.37 (12)
La4^iv^—La4—La4^v^	60.00 (10)	La4^xviii^—La9—Ru1^xv^	162.55 (18)
La4^iv^—La4—La5^i^	108.42 (14)	La4^xviii^—La9—Ru3^xv^	64.02 (13)
La4^iv^—La4—La5^ii^	59.41 (11)	La5^x^—La9—La5^xv^	62.04 (14)
La4^iv^—La4—La7^i^	109.16 (17)	La5^x^—La9—La6	149.48 (9)
La4^iv^—La4—La8^i^	149.58 (12)	La5^x^—La9—La6^xv^	109.92 (10)
La4^iv^—La4—La9^xi^	90.28 (9)	La5^x^—La9—La8^xxiv^	86.47 (14)
La4^iv^—La4—La9^viii^	146.03 (17)	La5^x^—La9—La9^x^	91.69 (14)
La4^iv^—La4—La10^xii^	61.51 (12)	La5^x^—La9—La9^xv^	60.93 (12)
La4^iv^—La4—Ru1^viii^	97.75 (15)	La5^x^—La9—Ru1	128.7 (2)
La4^iv^—La4—Ru1^xiii^	51.80 (12)	La5^x^—La9—Ru1^xv^	69.16 (16)
La4^iv^—La4—Ru5	48.17 (10)	La5^x^—La9—Ru3^xv^	47.12 (11)
La4^v^—La4—La5^i^	59.41 (10)	La5^xv^—La9—La6	109.92 (13)
La4^v^—La4—La5^ii^	108.42 (13)	La5^xv^—La9—La6^xv^	149.48 (12)
La4^v^—La4—La7^i^	109.16 (18)	La5^xv^—La9—La8^xxiv^	86.47 (15)
La4^v^—La4—La8^i^	149.58 (9)	La5^xv^—La9—La9^x^	60.93 (13)
La4^v^—La4—La9^xi^	146.03 (13)	La5^xv^—La9—La9^xv^	91.69 (14)
La4^v^—La4—La9^viii^	90.28 (11)	La5^xv^—La9—Ru1	69.16 (17)
La4^v^—La4—La10^xii^	61.51 (13)	La5^xv^—La9—Ru1^xv^	128.7 (2)
La4^v^—La4—Ru1^viii^	51.80 (15)	La5^xv^—La9—Ru3^xv^	47.12 (13)
La4^v^—La4—Ru1^xiii^	97.75 (14)	La6—La9—La6^xv^	60.69 (13)
La4^v^—La4—Ru5	48.17 (11)	La6—La9—La8^xxiv^	123.49 (12)
La5^i^—La4—La5^ii^	111.1 (2)	La6—La9—La9^x^	61.31 (13)
La5^i^—La4—La7^i^	61.92 (13)	La6—La9—La9^xv^	91.44 (14)
La5^i^—La4—La8^i^	93.44 (10)	La6—La9—Ru1	43.62 (16)
La5^i^—La4—La9^xi^	153.95 (16)	La6—La9—Ru1^xv^	103.7 (2)
La5^i^—La4—La9^viii^	62.49 (11)	La6—La9—Ru3^xv^	149.61 (12)
La5^i^—La4—La10^xii^	113.65 (12)	La6^xv^—La9—La8^xxiv^	123.49 (15)
La5^i^—La4—Ru1^viii^	71.20 (16)	La6^xv^—La9—La9^x^	91.44 (15)
La5^i^—La4—Ru1^xiii^	156.97 (13)	La6^xv^—La9—La9^xv^	61.31 (12)
La5^i^—La4—Ru5	63.93 (11)	La6^xv^—La9—Ru1	103.7 (2)
La5^ii^—La4—La7^i^	61.92 (13)	La6^xv^—La9—Ru1^xv^	43.62 (16)
La5^ii^—La4—La8^i^	93.44 (13)	La6^xv^—La9—Ru3^xv^	149.61 (13)
La5^ii^—La4—La9^xi^	62.49 (12)	La8^xxiv^—La9—La9^x^	143.40 (15)
La5^ii^—La4—La9^viii^	153.95 (16)	La8^xxiv^—La9—La9^xv^	143.40 (16)
La5^ii^—La4—La10^xii^	113.65 (10)	La8^xxiv^—La9—Ru1	105.97 (8)
La5^ii^—La4—Ru1^viii^	156.97 (13)	La8^xxiv^—La9—Ru1^xv^	105.97 (16)
La5^ii^—La4—Ru1^xiii^	71.20 (15)	La8^xxiv^—La9—Ru3^xv^	48.44 (13)
La5^ii^—La4—Ru5	63.93 (11)	La9^x^—La9—La9^xv^	60.00 (10)
La7^i^—La4—La8^i^	62.46 (14)	La9^x^—La9—Ru1	49.31 (9)
La7^i^—La4—La9^xi^	95.29 (14)	La9^x^—La9—Ru1^xv^	107.43 (15)
La7^i^—La4—La9^viii^	95.29 (12)	La9^x^—La9—Ru3^xv^	106.82 (19)
La7^i^—La4—La10^xii^	168.85 (15)	La9^xv^—La9—Ru1	107.43 (13)
La7^i^—La4—Ru1^viii^	131.07 (16)	La9^xv^—La9—Ru1^xv^	49.31 (14)
La7^i^—La4—Ru1^xiii^	131.07 (17)	La9^xv^—La9—Ru3^xv^	106.82 (18)
La7^i^—La4—Ru5	72.63 (15)	Ru1—La9—Ru1^xv^	144.2 (3)
La8^i^—La4—La9^xi^	63.11 (8)	Ru1—La9—Ru3^xv^	106.59 (19)
La8^i^—La4—La9^viii^	63.11 (8)	Ru1^xv^—La9—Ru3^xv^	106.6 (2)
La8^i^—La4—La10^xii^	128.7 (2)	La1—La10—La3	89.13 (16)
La8^i^—La4—Ru1^viii^	109.43 (14)	La1—La10—La3^iv^	89.13 (16)
La8^i^—La4—Ru1^xiii^	109.43 (15)	La1—La10—La3^v^	89.13 (16)
La8^i^—La4—Ru5	135.1 (2)	La1—La10—La4^xxv^	146.57 (9)
La9^xi^—La4—La9^viii^	111.09 (14)	La1—La10—La4^ix^	146.57 (9)
La9^xi^—La4—La10^xii^	91.00 (15)	La1—La10—La4^xviii^	146.57 (9)
La9^xi^—La4—Ru1^viii^	125.4 (2)	La1—La10—La6	72.72 (14)
La9^xi^—La4—Ru1^xiii^	48.44 (11)	La1—La10—La6^iv^	72.72 (14)
La9^xi^—La4—Ru5	124.08 (11)	La1—La10—La6^v^	72.72 (15)
La9^viii^—La4—La10^xii^	91.00 (15)	La1—La10—Ru1	119.21 (17)
La9^viii^—La4—Ru1^viii^	48.44 (11)	La1—La10—Ru1^iv^	119.21 (17)
La9^viii^—La4—Ru1^xiii^	125.4 (2)	La1—La10—Ru1^v^	119.21 (17)
La9^viii^—La4—Ru5	124.08 (13)	La3—La10—La3^iv^	119.98 (6)
La10^xii^—La4—Ru1^viii^	49.39 (13)	La3—La10—La3^v^	119.98 (9)
La10^xii^—La4—Ru1^xiii^	49.39 (14)	La3—La10—La4^xxv^	106.74 (16)
La10^xii^—La4—Ru5	96.21 (11)	La3—La10—La4^ix^	57.45 (14)
Ru1^viii^—La4—Ru1^xiii^	97.7 (2)	La3—La10—La4^xviii^	106.74 (16)
Ru1^viii^—La4—Ru5	99.50 (16)	La3—La10—La6	61.19 (7)
Ru1^xiii^—La4—Ru5	99.50 (9)	La3—La10—La6^iv^	161.8 (3)
La2^xvi^—La5—La2^xviii^	60.49 (13)	La3—La10—La6^v^	61.19 (7)
La2^xvi^—La5—La4^xvi^	156.03 (16)	La3—La10—Ru1	64.60 (10)
La2^xvi^—La5—La4^xix^	113.64 (11)	La3—La10—Ru1^iv^	151.7 (3)
La2^xvi^—La5—La7	106.2 (2)	La3—La10—Ru1^v^	64.60 (11)
La2^xvi^—La5—La7^xiv^	57.20 (11)	La3^iv^—La10—La3^v^	119.98 (8)
La2^xvi^—La5—La9^x^	142.72 (10)	La3^iv^—La10—La4^xxv^	106.74 (16)
La2^xvi^—La5—La9^xv^	107.83 (13)	La3^iv^—La10—La4^ix^	106.74 (17)
La2^xvi^—La5—Ru3	119.61 (19)	La3^iv^—La10—La4^xviii^	57.45 (14)
La2^xvi^—La5—Ru3^x^	61.67 (17)	La3^iv^—La10—La6	61.19 (7)
La2^xviii^—La5—La4^xvi^	113.64 (13)	La3^iv^—La10—La6^iv^	61.19 (9)
La2^xviii^—La5—La4^xix^	156.03 (17)	La3^iv^—La10—La6^v^	161.8 (3)
La2^xviii^—La5—La7	57.20 (12)	La3^iv^—La10—Ru1	64.60 (11)
La2^xviii^—La5—La7^xiv^	106.20 (19)	La3^iv^—La10—Ru1^iv^	64.60 (15)
La2^xviii^—La5—La9^x^	107.83 (10)	La3^iv^—La10—Ru1^v^	151.7 (3)
La2^xviii^—La5—La9^xv^	142.72 (11)	La3^v^—La10—La4^xxv^	57.45 (14)
La2^xviii^—La5—Ru3	61.67 (16)	La3^v^—La10—La4^ix^	106.74 (17)
La2^xviii^—La5—Ru3^x^	119.6 (2)	La3^v^—La10—La4^xviii^	106.74 (16)
La4^xvi^—La5—La4^xix^	61.19 (11)	La3^v^—La10—La6	161.8 (3)
La4^xvi^—La5—La7	58.39 (13)	La3^v^—La10—La6^iv^	61.19 (11)
La4^xvi^—La5—La7^xiv^	107.78 (11)	La3^v^—La10—La6^v^	61.19 (9)
La4^xvi^—La5—La9^x^	60.52 (11)	La3^v^—La10—Ru1	151.7 (3)
La4^xvi^—La5—La9^xv^	90.14 (18)	La3^v^—La10—Ru1^iv^	64.60 (15)
La4^xvi^—La5—Ru3	67.20 (13)	La3^v^—La10—Ru1^v^	64.60 (11)
La4^xvi^—La5—Ru3^x^	126.2 (2)	La4^xxv^—La10—La4^ix^	56.99 (12)
La4^xix^—La5—La7	107.78 (12)	La4^xxv^—La10—La4^xviii^	56.99 (13)
La4^xix^—La5—La7^xiv^	58.39 (12)	La4^xxv^—La10—La6	140.7 (2)
La4^xix^—La5—La9^x^	90.14 (17)	La4^xxv^—La10—La6^iv^	89.14 (12)
La4^xix^—La5—La9^xv^	60.52 (12)	La4^xxv^—La10—La6^v^	89.14 (12)
La4^xix^—La5—Ru3	126.2 (2)	La4^xxv^—La10—Ru1	94.2 (2)
La4^xix^—La5—Ru3^x^	67.20 (12)	La4^xxv^—La10—Ru1^iv^	49.63 (16)
La7—La5—La7^xiv^	109.14 (15)	La4^xxv^—La10—Ru1^v^	49.63 (15)
La7—La5—La9^x^	92.03 (13)	La4^ix^—La10—La4^xviii^	56.99 (14)
La7—La5—La9^xv^	145.8 (2)	La4^ix^—La10—La6	89.14 (12)
La7—La5—Ru3	51.08 (11)	La4^ix^—La10—La6^iv^	140.7 (2)
La7—La5—Ru3^x^	159.8 (2)	La4^ix^—La10—La6^v^	89.14 (13)
La7^xiv^—La5—La9^x^	145.81 (19)	La4^ix^—La10—Ru1	49.63 (15)
La7^xiv^—La5—La9^xv^	92.03 (12)	La4^ix^—La10—Ru1^iv^	94.2 (2)
La7^xiv^—La5—Ru3	159.8 (2)	La4^ix^—La10—Ru1^v^	49.63 (14)
La7^xiv^—La5—Ru3^x^	51.08 (11)	La4^xviii^—La10—La6	89.14 (12)
La9^x^—La5—La9^xv^	58.14 (13)	La4^xviii^—La10—La6^iv^	89.14 (13)
La9^x^—La5—Ru3	50.33 (16)	La4^xviii^—La10—La6^v^	140.7 (2)
La9^x^—La5—Ru3^x^	107.2 (2)	La4^xviii^—La10—Ru1	49.63 (13)
La9^xv^—La5—Ru3	107.2 (2)	La4^xviii^—La10—Ru1^iv^	49.63 (15)
La9^xv^—La5—Ru3^x^	50.33 (17)	La4^xviii^—La10—Ru1^v^	94.2 (2)
Ru3—La5—Ru3^x^	148.13 (17)	La6—La10—La6^iv^	111.57 (14)
La2^iv^—La6—La2^vii^	113.7 (2)	La6—La10—La6^v^	111.57 (13)
La2^iv^—La6—La3	155.60 (17)	La6—La10—Ru1	46.49 (15)
La2^iv^—La6—La3^iv^	60.62 (12)	La6—La10—Ru1^iv^	124.17 (15)
La2^iv^—La6—La7^i^	57.87 (13)	La6—La10—Ru1^v^	124.17 (9)
La2^iv^—La6—La9	89.74 (14)	La6^iv^—La10—La6^v^	111.57 (14)
La2^iv^—La6—La9^x^	143.08 (16)	La6^iv^—La10—Ru1	124.17 (12)
La2^iv^—La6—La10	106.29 (9)	La6^iv^—La10—Ru1^iv^	46.49 (15)
La2^iv^—La6—Ru1	122.97 (15)	La6^iv^—La10—Ru1^v^	124.17 (11)
La2^iv^—La6—Ru2	65.22 (12)	La6^v^—La10—Ru1	124.17 (10)
La2^vii^—La6—La3	60.62 (12)	La6^v^—La10—Ru1^iv^	124.17 (14)
La2^vii^—La6—La3^iv^	155.60 (17)	La6^v^—La10—Ru1^v^	46.49 (16)
La2^vii^—La6—La7^i^	57.87 (13)	Ru1—La10—Ru1^iv^	98.2 (2)
La2^vii^—La6—La9	143.08 (13)	Ru1—La10—Ru1^v^	98.2 (2)
La2^vii^—La6—La9^x^	89.74 (12)	Ru1^iv^—La10—Ru1^v^	98.2 (2)
La2^vii^—La6—La10	106.29 (12)	La4^ix^—Ru1—La4^xviii^	76.40 (18)
La2^vii^—La6—Ru1	122.97 (17)	La4^ix^—Ru1—La6	136.9 (2)
La2^vii^—La6—Ru2	65.22 (11)	La4^ix^—Ru1—La9	129.5 (3)
La3—La6—La3^iv^	113.60 (15)	La4^ix^—Ru1—La9^x^	80.19 (16)
La3—La6—La7^i^	106.48 (16)	La4^ix^—Ru1—La10	80.98 (19)
La3—La6—La9	108.29 (19)	La4^xviii^—Ru1—La6	136.93 (18)
La3—La6—La9^x^	60.69 (12)	La4^xviii^—Ru1—La9	80.19 (16)
La3—La6—La10	57.86 (8)	La4^xviii^—Ru1—La9^x^	129.5 (3)
La3—La6—Ru1	65.18 (17)	La4^xviii^—Ru1—La10	80.98 (15)
La3—La6—Ru2	123.03 (11)	La6—Ru1—La9	89.0 (2)
La3^iv^—La6—La7^i^	106.48 (14)	La6—Ru1—La9^x^	88.96 (18)
La3^iv^—La6—La9	60.69 (12)	La6—Ru1—La10	79.9 (2)
La3^iv^—La6—La9^x^	108.29 (19)	La9—Ru1—La9^x^	81.38 (16)
La3^iv^—La6—La10	57.86 (8)	La9—Ru1—La10	137.99 (16)
La3^iv^—La6—Ru1	65.18 (14)	La9^x^—Ru1—La10	138.0 (2)
La3^iv^—La6—Ru2	123.03 (14)	La6—Ru2—La6^x^	88.3 (2)
La7^i^—La6—La9	145.16 (14)	La6—Ru2—La6^xv^	88.3 (2)
La7^i^—La6—La9^x^	145.16 (9)	La6—Ru2—La7^i^	77.58 (15)
La7^i^—La6—La10	107.53 (17)	La6—Ru2—La7^xxvi^	133.93 (12)
La7^i^—La6—Ru1	161.16 (17)	La6—Ru2—La7^xiii^	133.93 (8)
La7^i^—La6—Ru2	53.30 (17)	La6^x^—Ru2—La6^xv^	88.3 (2)
La9—La6—La9^x^	57.38 (12)	La6^x^—Ru2—La7^i^	133.93 (12)
La9—La6—La10	92.76 (16)	La6^x^—Ru2—La7^xxvi^	77.58 (15)
La9—La6—Ru1	47.41 (16)	La6^x^—Ru2—La7^xiii^	133.93 (10)
La9—La6—Ru2	104.02 (16)	La6^xv^—Ru2—La7^i^	133.93 (9)
La9^x^—La6—La10	92.76 (17)	La6^xv^—Ru2—La7^xxvi^	133.93 (10)
La9^x^—La6—Ru1	47.41 (12)	La6^xv^—Ru2—La7^xiii^	77.58 (16)
La9^x^—La6—Ru2	104.02 (14)	La7^i^—Ru2—La7^xxvi^	81.4 (2)
La10—La6—Ru1	53.63 (17)	La7^i^—Ru2—La7^xiii^	81.4 (2)
La10—La6—Ru2	160.8 (3)	La7^xxvi^—Ru2—La7^xiii^	81.4 (2)
Ru1—La6—Ru2	145.5 (2)	La5—Ru3—La5^xv^	88.43 (13)
La1^xvi^—La7—La2^xx^	88.43 (12)	La5—Ru3—La7	80.05 (14)
La1^xvi^—La7—La2^xviii^	88.43 (12)	La5—Ru3—La7^vi^	139.0 (3)
La1^xvi^—La7—La4^xvi^	86.35 (15)	La5—Ru3—La8	132.81 (15)
La1^xvi^—La7—La5	70.25 (10)	La5—Ru3—La9^x^	82.55 (18)
La1^xvi^—La7—La5^xxi^	70.25 (10)	La5^xv^—Ru3—La7	139.0 (3)
La1^xvi^—La7—La6^xvi^	71.13 (16)	La5^xv^—Ru3—La7^vi^	80.05 (14)
La1^xvi^—La7—La7^vi^	145.15 (15)	La5^xv^—Ru3—La8	132.81 (15)
La1^xvi^—La7—La7^vii^	145.15 (14)	La5^xv^—Ru3—La9^x^	82.55 (17)
La1^xvi^—La7—La8	145.7 (2)	La7—Ru3—La7^vi^	83.41 (17)
La1^xvi^—La7—Ru2^xvi^	120.3 (2)	La7—Ru3—La8	79.27 (14)
La1^xvi^—La7—Ru3	119.07 (13)	La7—Ru3—La9^x^	133.59 (16)
La1^xvi^—La7—Ru3^vii^	119.07 (9)	La7^vi^—Ru3—La8	79.27 (15)
La2^xx^—La7—La2^xviii^	121.6 (2)	La7^vi^—Ru3—La9^x^	133.59 (17)
La2^xx^—La7—La4^xvi^	118.98 (13)	La8—Ru3—La9^x^	81.9 (2)
La2^xx^—La7—La5	158.60 (12)	La1—Ru4—La2	126.9 (2)
La2^xx^—La7—La5^xxi^	61.46 (12)	La1—Ru4—La2^vii^	126.9 (2)
La2^xx^—La7—La6^xvi^	61.97 (15)	La1—Ru4—La3	130.3 (3)
La2^xx^—La7—La7^vi^	108.92 (13)	La2—Ru4—La2^vii^	84.25 (16)
La2^xx^—La7—La7^vii^	56.73 (10)	La2—Ru4—La3	86.7 (2)
La2^xx^—La7—La8	107.30 (13)	La2^vii^—Ru4—La3	86.69 (18)
La2^xx^—La7—Ru2^xvi^	65.98 (12)	La1—Ru5—La4	129.64 (11)
La2^xx^—La7—Ru3	152.50 (14)	La1—Ru5—La4^iv^	129.64 (11)
La2^xx^—La7—Ru3^vii^	63.25 (15)	La1—Ru5—La4^v^	129.64 (11)
La2^xviii^—La7—La4^xvi^	118.98 (15)	La4—Ru5—La4^iv^	83.65 (12)
La2^xviii^—La7—La5	61.46 (12)	La4—Ru5—La4^v^	83.65 (13)
La2^xviii^—La7—La5^xxi^	158.60 (14)	La4^iv^—Ru5—La4^v^	83.65 (14)
La2^xviii^—La7—La6^xvi^	61.97 (14)		

Symmetry codes: (i) −*x*+1, −*y*+1, *z*+1/2; (ii) *y*+1, −*x*+*y*+1, *z*+1/2; (iii) *x*−*y*+1, *x*+1, *z*+1/2; (iv) −*y*+2, *x*−*y*+1, *z*; (v) −*x*+*y*+1, −*x*+2, *z*; (vi) −*y*+1, *x*−*y*+1, *z*; (vii) −*x*+*y*, −*x*+1, *z*; (viii) *y*, −*x*+*y*+1, *z*+1/2; (ix) *y*, −*x*+*y*+1, *z*−1/2; (x) −*y*+1, *x*−*y*, *z*; (xi) −*x*+2, −*y*+1, *z*+1/2; (xii) −*x*+2, −*y*+2, *z*+1/2; (xiii) *x*−*y*+1, *x*, *z*+1/2; (xiv) −*x*+*y*, −*x*, *z*; (xv) −*x*+*y*+1, −*x*+1, *z*; (xvi) −*x*+1, −*y*+1, *z*−1/2; (xvii) *x*−1, *y*, *z*; (xviii) *x*−*y*+1, *x*, *z*−1/2; (xix) *x*−*y*, *x*−1, *z*−1/2; (xx) *y*−1, −*x*+*y*, *z*−1/2; (xxi) −*y*, *x*−*y*, *z*; (xxii) *x*−*y*, *x*, *z*−1/2; (xxiii) −*x*+2, −*y*+1, *z*−1/2; (xxiv) *x*+1, *y*, *z*; (xxv) −*x*+2, −*y*+2, *z*−1/2; (xxvi) *y*, −*x*+*y*, *z*+1/2.
